# Dentists’ Knowledge and Treatment Preferences on Dental Erosive Lesions in Southern India: Implications for Policy and Education

**DOI:** 10.1155/ijod/7611231

**Published:** 2026-08-30

**Authors:** Ishani Sengupta, Krishnaraj Somayaji, Prajna Pramod Nayak, Spoorthi S. Shetty, Anwar Khan P., Kartikeya Bolar

**Affiliations:** ^1^ Department of Conservative Dentistry and Endodontics, Manipal College of Dental Sciences, Manipal Academy of Higher Education, Manipal, India, manipal.edu; ^2^ Department of Public Health Dentistry, Manipal College of Dental Sciences, Manipal Academy of Higher Education, Manipal, India, manipal.edu; ^3^ Manipal College of Dental Sciences, Manipal Academy of Higher Education, Manipal, India, manipal.edu; ^4^ Information Systems and Analytics, T A Pai Management Institute, Manipal Academy of Higher Education, Manipal, India, manipal.edu

**Keywords:** dental erosion, dental treatment, diagnosis, erosive tooth wear, private dental practitioners

## Abstract

**Background:**

Early diagnosis and proper treatment are essential for dental erosion (DE), a condition that is gaining more recognition in recent years. This study surveyed dentists in Southern India to assess their knowledge of DE, diagnostic approaches, and treatment preferences.

**Methods:**

We used a self‐administered questionnaire (*n* = 110) aimed at assessing demographics, frequency of DE encounter, perceived prevalence trends, etiological understanding, preventive advice, and preferred treatments. Descriptive statistics, chi‐square test of association, multivariate logistic regressions, and clustering analysis were conducted.

**Results:**

The sample consisted of 71% females and mean age of 31.46 ± 4.29 years, had predominantly learned about DE in dental schools (85%). Upper incisors were identified as most frequently affected teeth (72%). Most of the preventive counseling focused on reducing acidic intake (58%). No significant association was found between the years of experience and frequency of DE cases seen (*p* = 0.68). Multinomial regression of treatment choices indicated that specialists pursued invasive restorations like ceramic veneers odds ratio (OR) = 5.42 (95% CI: ~1.23−24.0, *p* = 0.025) or crowns (95% CI: ~1.12−14.3, *p* = 0.034) more than the general dentists, whereas higher years of experience was associated with a lower probability of choosing mid‐level restorative options (*p*  < 0.05). Clustering analysis revealed three practitioner profiles: (1) an “Aggressive/Referral” cluster of mostly younger dentists who frequently planned crowns and referred cases frequently (67% referral rate), (2) a “Conservative/Preventive” cluster of more experienced general dentists who emphasized monitoring and dietary advice with minimal operative interventions and infrequent referrals, and (3) an “Intermediate” cluster who implemented modern practices like index usage but also held more misconceptions.

**Conclusion:**

Dentists in Southern India exhibited a reasonable level of DE awareness. The need for clinical guidelines on erosive tooth wear and ongoing professional education is highlighted by the lower use of standardized indices and discrepancies in clinical management. Training in early detection, cause‐specific management of DE, and debunking myths will be necessary to improve patient outcomes in this developing field of dental care.

## 1. Introduction

Tooth wear is a complex process that results from the combined effects of biological, mechanical, chemical, and tribological influences, which interact to cause a gradual degradation of dental structures [1–3]. Dental erosion (DE), which is the chemical loss of mineralized tooth structure not originating from oral bacteria, has gained attention as a significant oral health issue over the past few years [1, 4]. High prevalences of erosive tooth wear have been reported, especially among younger populations worldwide [5–7]. The multifactorial etiology of DE includes extrinsic acids (foods and beverages) and intrinsic acids (gastric reflux and eating disorders) and is modified by saliva and oral hygiene practices. In clinical practice, distinguishing DE from other forms of tooth wear (attrition from bruxism and abrasion from aggressive brushing) is challenging but essential for appropriate management [8–10].

There is growing attention on DE from both the public and dental health professionals, potentially attributed to declining caries rates in some countries and the rising reporting of DE lesions in recent literature [6, 7, 11]. Patients may experience major functional, cosmetic, and psychological effects from the loss of intact dental tissues [12, 13]. This challenge is further complicated by limited recognition of the condition within the general population [12–14], which delays seeking treatment until it has progressed to a point where symptoms like dentinal hypersensitivity or the need for restorative procedures are evident.

Since identification mainly depends on clinical examination in the absence of commonly used early quantitative detection tools, clinicians need to be skilled in the clinical diagnosis of erosive lesions [15]. Smooth, concave defects are the initial appearance of these lesions, which then progress to cupping and, in more advanced stages, exposed dentin or flattened occlusal anatomy [4, 16]. The palatal/lingual surfaces of the maxillary anterior teeth are frequently affected by intrinsic acid, while the labial/facial surfaces are more frequently affected by extrinsic dietary acids. Maxillary incisors are often affected in gastric reflux and bulimia, while mandibular teeth may be comparatively shielded by the tongue and salivary pool [15–17]. When necessary, management usually includes minimally invasive restorative care, accurate diagnosis and investigation to treat underlying causes, patient education, preventive measures, and regular follow‐up.

Recent literature indicates that if risk factors are not addressed and preventive measures are not given timely, erosive tooth wear will continue to worsen [18, 19]. Dental professionals may fail to notice early tooth surface loss due to its subtle presentation and frequently painless early stages [20]. The lack of standardized application and documentation in routine practice still hinders consistent detection and epidemiological comparisons, even after indices like the Eccles Index and the Basic Erosive Wear Examination (BEWE) were introduced to measure tooth wear [8, 21].

The results of studies on recent clinical practices are not entirely consistent. A survey of Norwegian dentists found that most recorded erosive wear and used clinical photographs and study models for documentation, but fewer practitioners emphasized early prevention strategies or routinely assessed salivary function and dietary acids [8]. A Finnish study reported similar findings, where high levels of recording but limited use of detailed scoring systems, infrequent salivary assessment, and no major differences between general practitioners and specialists in treatment decisions—highlighting the need for standardized clinical guidance [9]. Studies from other settings (Yemen, Australia, and Jordan) and recent systematic reviews also indicate gaps in awareness and variable management approaches among dental students, practitioners, and faculty members [6, 7, 11, 13, 14, 22–24]. Collectively, these data show that many clinicians advise patients about DE only occasionally or rarely [7, 22, 25]. To the best of our knowledge, no published study has comprehensively evaluated the awareness and clinical management practices related to DE among Indian dental practitioners. Therefore, the present study was undertaken to address this important knowledge gap and provide baseline data from the Indian context.

Given the limited data on diagnostic knowledge and treatment choices among dental practitioners in a coastal district of Southern India with a population of ~4.7 million, this study aimed to assess awareness, diagnostic practices, and treatment approaches for DE. By examining knowledge and treatment strategies, we sought to identify gaps and inform improvements in clinical practice and preventive care for DE.

The other objectives were to examine any association between practitioners’ background (years of experience, qualification, and practice setting) and clinical practices (use of DE indices, diagnostic tools, and treatment choices), to identify predictors of specific practices (e.g., use of an index or restoration choice), and to identify practitioner profiles in the diagnosis and management of erosive tooth wear.

## 2. Materials and Methods

Study design and participants: this cross‐sectional online questionnaire survey targeted dental practitioners in the Coastal Karnataka districts (Udupi, Dakshina Kannada, and Uttara Kannada) of South‐Western India. Dentists with at least 5 years of clinical experience practicing within these districts were included to ensure that participants had sufficient independent clinical exposure and experience in managing noncarious tooth surface loss. A list of ~350 active practitioners was compiled from the local dental association records and regulatory council registers. Coastal Karnataka is served by a small number of dental practitioners, and since this was an online survey, which usually has a low or unpredictable response rate, sampling was not done. Instead, all eligible participants were included in the study. Invitations were distributed electronically to all via emails and/or WhatsApp with study information and an e‐consent form; respondents provided written electronic consent. The institutional ethics committee approved the study protocol.

Questionnaire: a self‐administered questionnaire was developed using items adapted from prior studies and tailored to local practice and language use. The questionnaire was developed by Mulic et al. [8] and later validated by Kangasmaa et al. [9] and Sartawi et al. [14].

**Figure 1:**
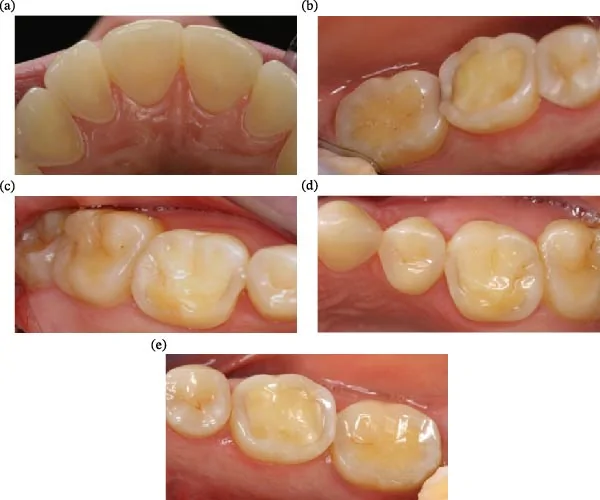
Clinical photographs of the patient case: a 28‐year‐old woman who had an eating disorder with vomiting as a teenager but is now healthy. (a) Palatal surfaces of the upper incisors, (b) occlusal surfaces of lower left 1st and 2nd molars, (c) occlusal surfaces of upper right 1st and 2nd molars, (d) occlusal surfaces of upper left 1st and 2nd molars, and (e) occlusal surfaces of lower right 1st and 2nd molars.

The instrument comprised four sections: (1) demographics including gender, age, year of graduation to compute experience, highest qualification general dentist (undergraduate dental qualification/BDS) and specialist (postgraduate qualification/MDS), and practice type (government/private/academic); (2) knowledge and perceptions of DE (sources of information, frequency of encountering DE, trends in prevalence, common surfaces, and etiologic beliefs—with true/false distractors included to gauge accuracy); (3) diagnostic and preventive practices (ability to recognize early signs, routine dietary enquiry, salivary assessment, and documentation practices); and (4) treatment decisions—featuring a clinical case scenario of moderate, generalized erosive wear in a patient with a brief patient history as well as color clinical photographs of labial and palatal surfaces of upper anterior and molar teeth was given. “Correct” or “incorrect” treatment decisions were not assumed, but responses were analyzed as preferences reflecting their clinical practice (Figure 1).

Participants were asked about advice they would want to give as well as their management with multiple‐choice options. Face and content validity were assessed qualitatively by subject experts. A pilot survey was conducted to confirm clarity and feasibility; minor wording refinements were made. Exploratory internal consistency across six dichotomous knowledge indicators yielded Cronbach’s *α* = 0.63; therefore, the questionnaire is treated as a multidomain instrument rather than a single psychometric scale.

Data collection: Google Forms was used to distribute the online survey between January and March of 2024. The raw data were transferred to Microsoft Excel for initial organization and then to Python (pandas) and SPSS v26 for analysis.

Statistical analysis: descriptive statistics were computed for all key variables. The proportion of dentists selecting each option for knowledge and practice questions were tabulated. For multiple‐response questions (e.g., causes, preventive advice), the percentage of respondents endorsing each item was calculated. Bivariate analyses were conducted to test associations as per the study objectives. Chi‐square (*χ* ^2^) tests were used for categorical comparisons, with significance at *p*  < 0.05. For multivariable analysis, two logistic regressions were performed. A binary logistic regression modeled the odds of a dentist using a formal DE grading index (yes/no) as the dependent variable, with independent predictors being qualification, years of experience, and practice sector. Also, multinomial logistic regression was used to explore factors influencing dentists’ choice of treatment intensity for the case scenario. To identify patterns in the responses, unsupervised learning techniques were applied. A hierarchical cluster analysis (Ward’s method) was performed on selected binary variables capturing the dentists’ diagnostic approach, preventive orientation, inclination to invasive treatment, and presence of any misconceptions.

## 3. Results

### 3.1. Demographic and Practice Profile

**Figure 2:**
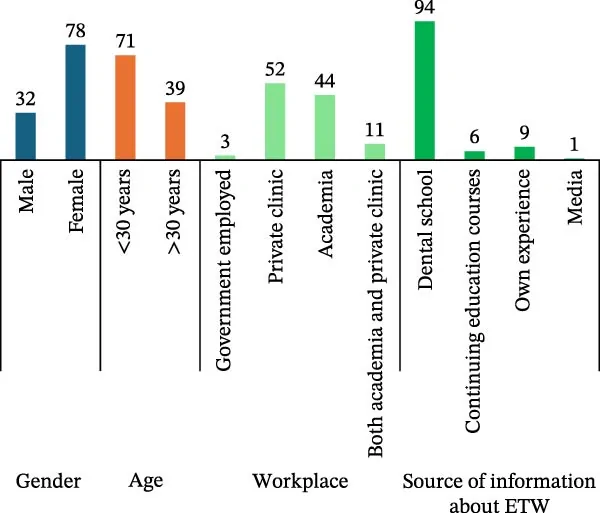
Distribution of dentists according to gender, age, workplace, and source of information about DE.

A total of 122 dentists responded to the survey, with 12 indicating that they did not work with DE or had less than 5 years of practice experience. As a result, 110 dentists participated (31% response rate). The majority (71%) of respondents were women, and their ages ranged from 28 to over 60 years, with a mean age of 31.46 ± 4.29 years. In terms of practice setting, 51 dentists (45%) worked primarily in private practice, 44 (39%) in academic institutions (dental colleges/hospitals), and only 3 (2.7%) in government dental services. An additional 15 practitioners (13%) indicated mixed roles: 11 split time between academia and private practice and 2 were engaged in both government and private practice. For analysis, those with mixed roles were considered based on the time devoted to an appropriate category. More than three‐fourths (85.4%) of participants’ main source of information about DE and its treatment was dental schools (Figure 2).

Table 1 shows any association of qualification and age with the practices of DE among participants. More than three‐fourths (83.4%) of participants agreed that the prevalence of DE has increased in the past 5 years. Most of the practitioners, irrespective of their qualification and age, agreed on the increasing prevalence of DE recently. Even though many dentists saw patients with DE frequently, there was no significant difference among graduates and postgraduates or their age. During the diagnosis of DE, most of them depended on clinical signs rather than an index for treatment planning. But there were no statistically significant differences among graduate and postgraduate practitioners as well as younger and older dentists (*p* = 0.133 and 0.090, respectively).

**Table 1:** Frequencies and percentages (%) of experience and practices on DE among participants.

Frequency		Qualification		Chi‐square *p*‐Value	Age		Chi‐square *p*‐Value
		General dentists (%)	Specialists (%)		<30 years (%)	>30 years (%)	
How often do you see patients with DE in your practice	Daily	73.7	26.3	2.348 0.672	52.6	47.4	2.019 0.732
	Weekly	58.5	41.5		68.3	31.7	
	Monthly	65.5	34.5		69.0	31.0	
	Yearly	55.6	44.4		55.6	44.4	
	Seldom	50.0	50.0		66.7	33.3	
I think the prevalence of DE has	Increased in past 5 years	63.9	36.1	1.240 0.538	68.7	31.3	2.854 0.240
	Decreased in past 5 years	42.9	57.1		42.9	57.1	
	Not changed	60.0	40.0		55.0	45.0	
During the diagnosis of DE	Depend on clinical signs	58.1	41.9	2.260 0.133	60.5	39.5	2.868 0.090
	Use index to grade severity	75.0	25.0		79.2	20.8	

Table 2 gives the participant’s knowledge of DE diagnosis, clinical features, and treatment. Regarding the most frequently affected areas, a significantly higher proportion of postgraduates answered correctly as compared to the graduates (*p* value = 0.018). Regarding the knowledge on acidic drinks as the main cause of DE, 81% and 69.1% of postgraduates and graduates, respectively, answered precisely. Whereas, regarding systemic diseases (e.g., eating disorders, stomach reflux) as other causes for DE, a significantly higher proportion of postgraduates answered correctly as compared to the graduates (*p* value = 0.005). However, a greater number of graduates believed that asking about dietary habits will help in the diagnosis and treatment plan of the case (*p* value = 0.025). Similarly, knowledge on DE was similar in both age groups except their belief on the need of dietary in the diagnosis, where younger participants stressed the need (*p* value = 0.018).

**Table 2:** Participant’s knowledge of the DE diagnosis, clinical features, and treatment.

Knowledge related questions	Qualification		Chi‐square *p*‐Value	Age		Chi‐square *p*‐Value
	General dentists (%)	Specialists (%)		<30 years (%)	>30 years (%)	
Tooth surface most affected	48.5	71.4	5.564 0.018 ^∗^	57.7	56.4	0.018 0.892
Main cause of DE	69.1	81.0	1.873 2171	71.8	76.9	0.336 0.562
Other causes of DE	60.3	85.7	7.989 0.005 ^∗^	71.8	66.7	0.320 0.572
Main clinical signs	77.9	78.6	0.006 0.938	81.7	71.8	1.445 0.229
Need for diet history	100.0	92.9	4.993 0.025 ^∗^	100.0	92.3	5.615 0.018 ^∗^
Most important advice for patients	66.2	73.8	0.708 0.400	69.0	69.2	0.001 0.981
Additional preventive measures	55.9	52.4	0.128 0.720	53.5	56.4	0.085 0.771

^∗^ *p* value  < 0.05.

When asked what type of advice they would give to the patient, 92.7% of participants stated that they provide information about dietary and drinking habits, followed by 52.7% referring to a specialist, faculty clinic, or other dentist and 45.5% recommending rinsing with fluoride. The lowest number of participants recommended rinsing with chlorhexidine (2.7%) (Table 3).

**Table 3:** The frequency (%) of dentists’ general patient advice(s) in the patient case.

Patient advice	Total (%)	Qualification		Age	
		General dentists (%)	Specialists (%)	<30 years (%)	>30 years (%)
Information about dietary and drinking habits	92.7	97.1	85.7	97.2	84.6
Information about brushing technique/habits	57.3	60.3	52.4	60.6	51.3
Recommend rinsing with fluoride	45.5	42.6	50.0	45.1	46.2
Recommend rinsing with chlorhexidine	2.7	2.9	2.4	4.2	0.0
Recommend fluoride tablets	13.6	13.2	14.3	15.5	10.3
Refer to specialist	52.7	52.9	52.4	57.7	43.6
Recommend specific toothpaste or rinse	34.9	34.3	35.7	27.1	48.7

*Note:* More than one choice was possible and values in the table denote the frequency of dentists’ advice expressed as percent.

Regarding how they would treat the maxillary anterior teeth and posterior teeth, more than 50% of participants stated that they would treat locally with fluoride solution, followed by 33.6% preferring to apply flowable composites and 29.1% preferring to apply bonding material. Restoring with compomers (2.7%) and glass ionomer cement (10%) were the least preferred by the practitioners (Table 4).

**Table 4:** The frequency (%) of dentists’ general choice of treatment(s) in the patient case.

Type of treatment preferred	Total (%)	Qualification		Age	
		General dentists (%)	Specialists (%)	<30 years (%)	>30 years (%)
No treatment	6.4	7.4	4.8	9.9	0.0
Treat locally with fluoride solution	53.6	50.0	59.5	56.3	48.7
Apply bonding material	29.1	29.4	28.6	29.6	28.2
Apply flow composite	33.6	35.3	31.0	35.2	30.8
Restore with glass ionomer cement	10.0	11.8	7.1	12.7	5.1
Restore with composite filling material	27.3	32.4	19.0	31.0	20.5
Restore with compomer	2.7	2.9	2.4	2.8	2.6
Restore with ceramic laminate/facet/inlay/onlay	27.3	26.5	28.6	26.8	28.2
Restore with crown	16.4	13.2	21.4	14.1	20.5

*Note:* More than one choice was possible and the values in the table denote the frequency of dentists’ choice of treatment expressed as percent.

The multinomial analysis for treatment choice with “no invasive treatment” as the baseline provided more insight. It revealed that dentist qualification was a significant predictor for choosing higher levels of treatment. Specifically, compared to doing nothing, the odds ratio (OR) of specialists (vs. general dentists) choosing to perform ceramic veneers/onlays was OR = 5.42 (95% CI: ~1.23−24.0, *p* = 0.025). Likewise, a specialist was about 4.02 times as likely as a general dentist to choose crowns over no treatment (95% CI: ~1.12−14.3, *p* = 0.034). But qualification did not significantly differentiate choosing moderate treatments (level 3 composite fillings, *p* = 0.089; level 2 flowable composites). The experience showed an inverse association with opting for certain treatments. Each additional year of experience reduced the log‐odds of choosing ceramic veneers (level 4) vs. none (coef = −0.144, *p* = 0.021), translating to about 15% decrease in odds per year, suggesting that older dentists were less likely to do ceramic rehabilitations. Experience was not a significant factor for crown choice (*p* = 0.12), implying that both younger and older clinicians would use crowns if needed.

In the exploratory binary logistic regression, none of the examined predictors (qualification, years since graduation, and practice setting) were significantly associated with using a formal diagnostic index. The estimated OR for specialists versus general dentists was 0.47 (95% CI 0.17–1.37, *p* = 0.168). Because only 24 participants reported index use, these multivariable estimates should be interpreted cautiously due to low events per variable and consequently wide confidence intervals (Table S1).

Clustering and practitioner profiles: three separate dentist clusters were found using hierarchical cluster analysis on important practice variables. Hierarchical clustering (Ward linkage) was performed on a prespecified set of binary variables representing the diagnostic approach and management preferences. To document robustness, we computed the average silhouette width for *k* = 3, which was ~0.15 (*k* = 2–5 range: 0.14–0.15), indicating weak separation. Therefore, clusters are presented as exploratory practitioner profiles rather than definitive latent classes (Table S2). These clusters can be described as various “profiles” of erosive wear management:

• Cluster 1: high referral tendencies and aggressive interveners—about 46% of the sample was made up of this cluster. For the case scenario, they decided on extensive treatments like crowns or veneers. This cluster had the highest referral rate among clusters, with 67% of respondents saying that they would refer the patient to a specialist. Merely 25% of them had a moderate understanding of the etiology and used an index.
• Cluster 2: conservative prevention‐oriented minimalists—making up ~36% of the sample, this cluster focused on preventive measures (diet advice and fluoride) and monitoring. Only 15% referred the case—they largely would manage it themselves. They showed very few misconceptions (only 7% selected any incorrect cause) and rarely suggested irrelevant prevention (only 9.8% gave night guard advice). Demographically, this cluster included more experienced dentists (mean ~6.9 years).
• Cluster 3: intermediate mixed‐strategy dentists with misconceptions—the smallest cluster (~18%,) exhibited a mixture of traits. They tended to do some restorative work (often composite fillings or, at most, partial restorations). 40% of them referred cases, 30% used an index—notably the highest index usage among clusters but had a relatively high level of misconceptions, and 40% recommended a night guard or similar misguided preventive measure. Their experience level (~5.6 years) and specialist percentage (~35%) were intermediate, representing younger general dentists.

**Figure 3:**
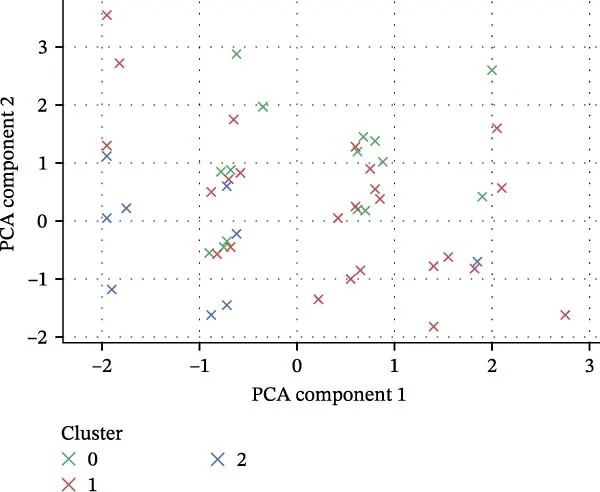
Cluster diagram of practitioners based on treatment preferences.

Figure 3 provides a dendrogram visualization of these clusters, illustrating the separation of the conservative vs. aggressive tendencies.

## 4. Discussion

The present study gains additional importance because although several Indian studies have documented the prevalence and distribution of DE among different population groups, there is limited evidence regarding how Indian dental practitioners diagnose and manage this condition. To the best of our knowledge, the present study is the first to comprehensively investigate these aspects in an Indian dental setting [25–27].

The fact that many dentists who studied DE in dental school can accurately identify dietary acids as the primary cause is encouraging. It implies that the dental curriculum has effectively taught basic concepts related to erosion. Nonetheless, we discovered enduring misconceptions among a subset of dentists, which is in line with previous surveys. More than 30% mistakenly believed that erosion was caused by bruxism and abrasion, which is similar to the trends observed in Yemen (41%), Jordan, and other countries [8, 11, 14]. The dentist may prescribe a night guard instead of looking into dietary habits or gastrointestinal issues if an erosive lesion is mistakenly attributed to bruxism. Advanced training improves theoretical knowledge, as evidenced by the fact that specialists were significantly more likely than general dentists to correctly identify uncommon intrinsic causes (such as reflux or eating disorders) (*p*  < 0.01).

With regards to diagnostic practices, low usage of any formal index (23%) aligns with Al‐Ashtal et al., who reported 27% index use, and Kangasmaa et al. [9] who found only 4% always use a detailed scoring system [11]. Time constraints, unfamiliarity, or the belief that indices (like BEWE) are unnecessary tools for everyday practice are some of the reasons why people are reluctant to use them. However, using a simple index can significantly improve the tracking of a patient’s erosive wear development and treatment results. Incorporating the BEWE or a similar tool into student clinics may change practice norms.

Early recognition is another diagnostic component. Very few dentists searched for advanced or inaccurate signs (shortened teeth, etc.), even though the majority of them could recognize the classic early sign (smooth, softened enamel). This emphasizes the necessity of additional clinical exposure or visual aids that show early erosion [28].

The general opinion among dentists regarding the etiology of DE is a positive finding and is consistent with data from around the world that show that between half and two‐thirds of dentists recommend diet changes. Additionally, our survey indicates that many dentists employ preventive strategies, such as fluoride treatments and patient education, either at home or in the office. However, the fact that a sizable percentage (19%) suggested night guards raises concerns because it suggests that dentists might be misdiagnosing DE as bruxism. It highlights again how important it is to distinguish erosion from other types [28].

Pertaining to treatment strategies, there was inconsistency among dentists in deciding on treatment options for the same situation. This reflects the lack of agreement on how to treat moderate erosive wear or the ambiguity of the evidence. Some dentists followed a minimally invasive philosophy which essentially was a preventive and additive approach (fluoride, bonding, and monitoring until the wear advances enough to justify restorations). Others took an interventive approach—restoring form and function early to protect teeth and satisfy esthetics or sensitivity concerns. In a study conducted by Al‐Ashtal et al. [11] in Yemen, 51% advised reducing acidic drinks and 37% advised fluoride toothpaste. Our study reported slightly higher fluoride recommendations and dietary advice, which may reflect increased global emphasis on noncarious lesions in the past decade. Kangasmaa et al. [9] reported very high fluoride application rates (80.9%) among Finnish dentists than those observed in the present study. This difference may reflect variations in undergraduate education, availability of standardized erosion management protocols, and differences in healthcare delivery between Finland and India. Finnish participants also included dentists practicing within a well‐established preventive oral healthcare system, which may partly explain the greater emphasis on topical fluoride therapy [9].

It is worth noting that current best practice for managing erosive wear tends to favor conservative measures initially, especially if the defects are not yet severe (enamel only or shallow dentin). This includes dietary management, fluoride, desensitization, and simple adhesive protections, all of which many of our respondents chose. Definitive restorations like crowns are usually delayed [2, 5, 24, 29]. The fact that one‐third of the advocated crowns in a moderate case might be on the higher side of intervention. Crown advocacy is usually favored by prosthodontists, for whom crowning teeth is a routine solution. It may also reflect that our “case scenario” description was interpreted by some as being advanced.

When we look at specialist vs. general dentist approaches, we found that specialists had roughly 4–5 times higher odds of planning an extensive restorative solution for erosive teeth rather than opting for monitoring, even after controlling for experience and practice type. This could be because specialists are trained to rehabilitate worn dentitions comprehensively, and they may lean toward that even in moderately worn cases to prevent further deterioration. Meanwhile, general dentists were more inclined to postpone extensive work, perhaps due to less familiarity with full‐mouth rehabilitation.

Cluster diagram showing practitioner profiles based on their treatment preferences helped in segregating the practitioners into 3 clusters: aggressive interveners, conservative prevention‐oriented minimalists, intermediate mixed‐strategic dentists with misconceptions. Although these exploratory practitioner profiles should not be interpreted as definitive classifications because of the weak cluster separation, they provide preliminary insights into variations in diagnostic and treatment approaches. Future studies with larger samples and external validation are required before these profiles can be used to guide targeted educational interventions. Nevertheless, regular screening for erosive wear should be included in dental exams, just like with caries and periodontal health.

Our data indicate that a large number of dentists are aware of the problem, though they deal with it infrequently. The prevalence is increasing due to the dietary shifts, particularly the growing consumption of soft drinks and acidic beverages. To counter this trend, patient education should focus on simple, practical steps such as cutting down the frequency of carbonated drinks and replacing acidic sports beverages with water or milk. Above all, dentists should aim to personalize treatment and prevention strategies, considering each patient’s lifestyle, diet, and level of risk.

Those practitioners with at least 5 years’ experience were included, yet many respondents were close to that cutoff, which could be due to easier online reach among younger dentists. The sample over‐represents younger and female dentists, which may reflect differential participation in online surveys and may bias observed practice patterns. Generalisability should therefore be cautious. Although the questionnaire was piloted and reviewed for clarity, face/content appropriateness, and exploratory internal consistency, it was not reliability‐tested, which may affect measurement precision and introduce response bias. As a cross‐sectional survey, the study identifies associations but cannot establish causality. Several multivariable analyses were exploratory. Particularly, index use occurred in very few respondents, resulting in low events per variable and wider confidence intervals; therefore, regression estimates should be interpreted cautiously. Additionally, our clustering analysis, while insightful, showed that the clusters were not predefined and could vary with different variable selections or methods. Also, self‐reported practices might differ from actual clinical behavior due to respondent bias or idealized answers. In addition, multinomial logistic regression used “no invasive treatment” as the reference category. As only a small proportion of participants (6.4%) selected this option, the resulting ORs may be unstable and associated with wide confidence intervals. Therefore, these findings should be interpreted as exploratory rather than confirmatory. Nonetheless, consistency with other studies lends credibility to the findings.

Recommendations: based on the findings, we propose the following:

1. Education and guidelines: the regional dental associations should create clinical practice guidelines for managing erosive tooth wear that address risk factor assessment, the use of baseline and monitoring indices, suggested intervention thresholds, and step‐by‐step treatment options (from adhesive restorations to prosthetic rehabilitation to fluoride therapy). Such guidelines will serve as a reference for dentists, lowering the uncertainty and variability of their practices.
2. Continuing professional development: plan seminars and workshops that address early erosion detection, distinguish between erosion, attrition, and abrasion, and dispel myths about contemporary minimally invasive management.
3. Integration in routine exams: motivate dentists to include an erosion check in their regular examinations.
4. Patient education materials: prepare pamphlets or online resources that teach patients how to stop DE. Having standardized materials can help ensure that important points are communicated, especially since dentists are recommending dietary changes [30].

## 5. Conclusion

The survey concludes by pointing out that although dentists in Southern India are adequately prepared to identify and prevent DE, there is a potential to standardize and improve their methods of diagnosis and care. Most practitioners take a proactive approach for early management using preventive agents like fluoride and counseling patients on dietary changes. Significant differences and gaps were also found by the survey, though a sizable minority mistake erosion for mechanical wear factors, objective diagnostic indices are still not widely used, and clinical management approaches vary widely from very conservative to very aggressive, suggesting a lack of standardized protocol. By developing clear clinical guidelines and strengthening educational initiatives focused on erosive tooth wear, the dental community can ensure more consistent and evidence‐based care. With erosion likely to become more prominent as a clinical problem, it is time to enhance professional training and develop consensus management pathways.

## Funding

The authors declare that no funding was involved in supporting this work.

## Conflicts of Interest

The authors declare no conflicts of interest.

## Supporting Information

Additional supporting information can be found online in the Supporting Information section.

## Supporting information


**Supporting Information** Table S1: Binary logistic regression predicting use of a formal dental erosion index (yes/no). None of the examined predictors (qualification, years since graduation, practice setting) were significantly associated with using a formal diagnostic index. Table S2: Cluster analysis specification and robustness indicators. Three separate dentist clusters were found using hierarchical cluster analysis on important practice variables.

## Data Availability

The datasets used and/or analyzed during the current study are available from the corresponding author upon reasonable request.
