# Supplementary material for: Dentists’ Knowledge and Treatment Preferences on Dental Erosive Lesions in Southern India: Implications for Policy and Education
Source: Int J Dent. 2026 Aug 30;2026:7611231. doi: 10.1155/ijod/7611231 (PMC13527397; doi:10.1155/ijod/7611231)
Supplement: Supplementary file 1 [file IJOD-2026-7611231-s001.doc]

Supplementary Tables:

Supplementary Table 1: Binary logistic regression predicting use of a formal dental erosion index (yes/no).

| Predictor | Contrast | Adjusted OR | 95% CI (low) | 95% CI (high) | p-value |
| --- | --- | --- | --- | --- | --- |
| Qualification | Specialists vs General dentists | 0.47 | 0.17 | 1.37 | 0.168 |
| Experience (years since graduation) | Per additional year | 0.98 | 0.91 | 1.06 | 0.633 |
| Practice setting | Academia vs Private | 1.07 | 0.38 | 2.97 | 0.900 |
|  | Both academia + private vs Private | 1.54 | 0.34 | 7.09 | 0.577 |
|  | Government vs Private | 1.92 | 0.16 | 23.81 | 0.611 |

Supplementary Table 2: Cluster analysis specification and robustness indicators

| Component | Specification / results |
| --- | --- |
| Clustering objective | Identify patterns (“profiles”) in dentists’ diagnostic and management choices (exploratory). |
| Algorithm | Agglomerative hierarchical clustering using Ward’s minimum-variance linkage on Euclidean distances (binary-encoded variables). |
| Variables included (binary) | Index use; referral; fluoride solution; flowable composite; ceramic laminate/facet/inlay/onlay; crown; knowledge on affected surface; knowledge on other causes. |
| Number of clusters | Three clusters selected from dendrogram inspection (Ward linkage). |
| Validation statistic | Average silhouette width ≈ 0.15 for k=3 (k=2–5 range: 0.14–0.15). |
